# Disproportionality analysis of upadacitinib-related adverse events in inflammatory bowel disease using the FDA adverse event reporting system

**DOI:** 10.3389/fphar.2025.1436183

**Published:** 2025-02-11

**Authors:** Shiyi Wang, Xiaojian Wang, Jing Ding, Xudong Zhang, Hongmei Zhu, Yihong Fan, Changbo Sun

**Affiliations:** ^1^ Department of Gastroenterology, Ningbo Hospital of Traditional Chinese Medicine, Affiliated to Zhejiang Chinese Medical University, Ningbo, China; ^2^ Department of Gastroenterology, Ningbo Medical Center LiHuiLi Hospital, Ningbo, China; ^3^ Department of Gastroenterology, First Affiliated Hospital of Zhejiang Chinese Medical University, Hangzhou, China

**Keywords:** upadacitinib, adverse event, FAERS, pharmacovigilance, data mining

## Abstract

**Background:**

Upadacitinib, a Janus kinase inhibitor, has been increasingly used over the past few years to treat moderate to severe ulcerative colitis and Crohn’s disease in patients who are insufficiently responsive or intolerant to tumor necrosis factor (TNF) antibodies, demonstrating notable clinical efficacy. The long-term safety of upadacitinib in extensive populations remains unexplored. This study evaluates upadacitinib-related adverse events (AEs) utilizing data from the US Food and Drug Administration Adverse Event Reporting System (FAERS).

**Methods:**

We employed disproportionality analyses, including the proportional reporting ratio (PRR), reporting odds ratio (ROR), Bayesian confidence propagation neural network (BCPNN), and empirical Bayesian geometric mean (EBGM) algorithms to identify signals of upadacitinib-associated AEs for treating inflammatory bowel disease (IBD).

**Results:**

From a total of 7,037,004 adverse event reports sourced from the FAERS database, 37,822 identified upadacitinib as the primary suspect drug in adverse drug events (ADEs), including 1,917 reports specifically related to the treatment of inflammatory bowel disease (IBD). The most commonly reported AEs were acne, product residue present, haematochezia, frequent bowel movements, flatulence, blood cholesterol increased, aligning with clinical trial outcomes. Notably, significant but unexpected AEs, such as rosacea, proctalgia, polyp, were also reported. Subgroup analysis indicated that the most prevalent AEs among the elderly included pulmonary embolism, cataract, and sepsis, whereas the 18–65 age group most frequently reported acne, abdominal pain, and nasopharyngitis. The median onset time for AEs related to upadacitinib was 41.00 days (interquartile range [IQR] 10–141.5 days), with the majority occurring within 3 months of treatment initiation (n = 269, 66.09%), particularly in the first month (n = 171, 42.01%).

**Conclusion:**

Our findings affirm clinical observations and reveal potential new AE signals for upadacitinib, underscoring the need for prospective clinical studies to verify these results and clarify their clinical relevance. This study contributes valuable evidence for ongoing safety evaluations of upadacitinib.

## 1 Introduction

Inflammatory Bowel Disease (IBD), primarily consisting of ulcerative colitis and Crohn’s disease, is classified as chronic, nonspecific inflammatory gastrointestinal disorders (Seyedian et al., 2019). Characterized by prolonged disease courses and recurrent conditions, IBD can lead to increased risks of surgery and disability as it progresses, consequently lowering the quality of life for patients and imposing greater economic burdens on families and society. While IBD is more commonly observed in younger populations (Kaplan and Windsor, 2021), it is particularly concerning that both the incidence and prevalence of IBD are on the rise among the elderly. Approximately 10%–15% of new IBD diagnoses occur in individuals aged 65 and above (elderly IBD). With the gradual aging of society, this percentage is expected to increase, presenting even greater challenges in the treatment and management of IBD (Singh et al., 2023; Sousa et al., 2023).

The etiology of IBD has not been fully elucidated, yet it is thought that genetic, immune, microbial, and environmental factors all contribute to the development and progression of the disease (Kofla-Dlubacz et al., 2022). Due to the insufficient efficacy and side effects of traditional drugs such as 5-aminosalicylic acid, corticosteroids, and immunosuppressants, some IBD patients do not achieve effective disease control. With a deeper understanding of the disease, advancements in diagnostic techniques, and the emergence of biologics, the therapeutic goals for IBD have shifted from merely alleviating clinical symptoms to achieving mucosal healing, aiming to reduce hospitalization, surgery rates, and achieve steroid-free remission (Turner et al., 2021). Antagonists of tumor necrosis factor (TNF) have been the longest-used and most widely applied, yet decades of data show a significant proportion of primary and secondary non-responders (Papamichael et al., 2019). Therefore, the continuous introduction of novel targeted therapies, such as small molecule drugs, has opened new paths for those poorly served by traditional treatments and TNF antagonist failures (Higashiyama and Hokari, 2023).

Upadacitinib, an oral selective JAK1 inhibitor, has been proven effective in treating IBD by inhibiting intestinal inflammation in multiple RCTs (Danese et al., 2022; Loftus et al., 2023). FDA has approved the drug for use in moderate to severe UC and CD patients who are insufficiently responsive or intolerant to TNF antibodies (RINVOQ^®^(upadacitinib)). Recent network meta-analyses have shown that upadacitinib surpasses most biologics in terms of endoscopic improvement during the induction phase, endoscopic remission, and histological remission during the maintenance phase (Shehab et al., 2024).

However, as with all pharmacological treatments, the use of upadacitinib carries a risk of adverse reactions. The FDA Adverse Event Reporting System (FAERS) is the largest publicly accessible, voluntary, spontaneous reporting database for drug monitoring worldwide (database; Sakaeda et al., 2013). This data is essential for evaluating the safety and efficacy of medications. Although the effectiveness of upadacitinib in treating IBD has been remarkably positive, extensive post-market empirical evidence is required to address ongoing debates and controversies over its safety. Consequently, our study aims to perform the first assessment of adverse event reports related to the treatment of IBD with upadacitinib from the third quarter of 2019 (Q3) to the first quarter of 2024 (Q1), with a specific focus on adverse events occurring in the elderly population.

## 2 Methods

### 2.1 Data source

Considering the timeline of the drug’s introduction to the market, this study downloaded the report files from the American Standard Code for Information Interchange (ASCII) FAERS database, available at https://fis.fda.gov/extensions/FPD-QDE-FAERS/FPD-QDE-FAERS.html, covering the period from the third quarter of 2019 to the first quarter of 2024. The FAERS database comprises seven categories of raw data: demographic and administrative information (DEMO), drug details (DRUG), adverse events (REAC), patient outcomes (OUTC), report sources (RPSR), start and end dates for reported drugs (THER), and indications for use (INDI). The data was imported into R version 4.3.2 for processing.

### 2.2 Data extraction and analysis

Duplicate reports were removed. For data in the DEMO table with the same CASEID, only the most recent report based on the date was retained. Relationships between datasets were established using the primaryid field, and anomalies in age and weight indicators were corrected. Drug names were standardized using the Medex_UIMA_1.8.3 system. To identify cases in the DRUG file, we used the drug’s brand and generic names (RINVOQ, upadacitinib) as keywords, with the main suspect (PS) used as the role code.

**FIGURE 1:**
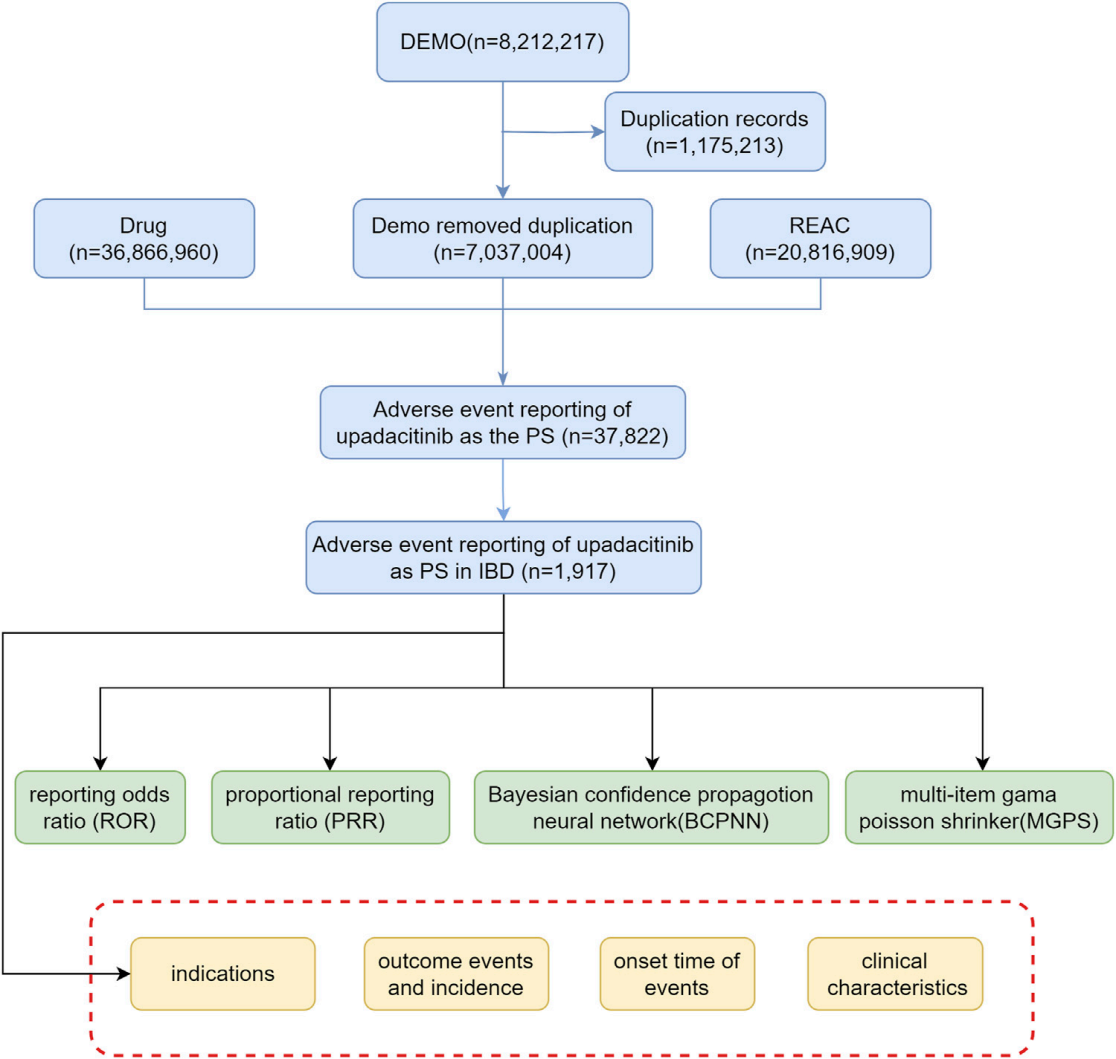
Flow diagram illustrating the selection of upadacitinib-related adverse events in IBD treatment from the FAERS database (Abbreviations: DEMO, demographic and administrative information; REAC, adverse event coding; PS, primary suspect drug).

Descriptive analyses were conducted to thoroughly examine reports of adverse events associated with upadacitinib, with a specific focus on clinical characteristics, including gender, age, reporting region, reporting time, and patient outcomes. Severe adverse patient outcomes were defined as hospitalization, disability, life-threatening, or death. Figure 1 illustrates a multi-step flowchart including data extraction, processing, and evaluation (Detailed data are presented in Supplementary Table S1).

### 2.3 Statistical analysis

In our study, we employed disproportionality measures commonly used in pharmacovigilance studies to detect potential signals between upadacitinib and adverse events (AEs). Disproportionality measures are widely used data mining methods globally, based on comparing frequency ratios observed in exposed and non-exposed populations using contingency tables to analyze the correlation between a drug and AEs (Noguchi et al., 2021) (Table 1). In this study, proportional reporting ratio (PRR), reporting odds ratio (ROR), bayesian confidence propagation neural network (BCPNN), and empirical bayesian geometric mean (EBGM) were used s (as shown in Table 2). The joint use of multiple algorithms allows for cross-validation to minimize false positives (Chen et al., 2012). Only those signals with at least three targeted drug-targeted AE records were calculated. A positive signal for drug-related adverse events should be considered if any of the four algorithms meet the criteria (lower limit of 95% CI > 1, N ≥ 3; PRR ≥2, χ^2^ ≥ 4, N ≥ 3; IC025 > 0 or EBGM05 > 2) (Zou et al., 2024). Considering that the risk of AEs in different age groups is not yet clear, subgroup analyses were conducted by age group (<18 years, 18–65 years, and >65 years). Statistical analysis was performed using R4.3.2 software. Higher values indicate stronger signal strength, suggesting a stronger association between the target drug and adverse events.

**TABLE 1:** Fourfold table of disproportionality method.

Medicine	Upadacitinib-related ADEs	Non-upadacitinib related ADEs	Total
Upadacitinib	a = 1917	b = 35,905	a + b = 37,822
Non-upadacitinib-	c = 232,598	d = 6,766,584	c + d = 6,999,182
Total	a+c = 234,515	b + d = 6,802,489	a + b + c + d = 7,037,004

**TABLE 2:** Methods, formulas, and thresholds for ROR, PRR, BCPNN, and EBGM.

Algorithms	Equation	Criteria
ROR	ROR = ad/b/c 95%CI = e^In (ROR) ± 1.96 (1/a + 1/b + 1/c + 1/d)^0.5^	Lower limit of 95% Cl > 1, N ≥ 3
PRR	PRR = a (c + d)/c/(a + b) χ^2^ = [(ad-bc)^2](a + b + c + d)/[(a + b) (c + d) (a+c) (b + d)]	PRR > 2, χ^2^ ≥ 4, N > 3
BCPNN	IC = log_2_a (a + b + c + d)/((a + c) (a + b)) 95%CI = E (IC) ± 2V (IC)^0.5	IC025 > 0
MGPS	EBGM = a (a + b + c + d)/(a + c)/(a + b) 95%CI = e^In (EBGM) ± 1.96 (1/a + 1/b + 1/c + 1/d)^0.5^	EBGM05 > 2

Notes: The contingency table for each pharmacovigilance algorithm includes: a, Reports with both the target drug and its specific adverse drug reaction. B, Reports featuring the target drug associated with different ADRs. c, Reports of the specific ADR linked to drugs other than the target. d, Reports documenting other drugs and unrelated ADRs.

Abbreviations: 95% Cl, 95% confidence interval; N, the number of reports; χ^2^, chi-squared; IC, information component; IC025, the lower limit of 95% CI of the IC; E (IC), the lC, expectations; V (IC), the variance of IC; EBGM, empirical Bayesian geometric mean; EBGM05, the lower limit of 95% CI Of EBGM.

### 2.4 Signal filtering and categorization

Using the latest version of the Medical Dictionary for Regulatory Activities terminology (MedDRA 25.0), we matched the preferred terms (PTs) and system organ classes (SOCs) for adverse reactions associated with upadacitinib. This allowed for encoding, categorization, and localization of signals to analyze specific SOCs involved in adverse event signals.

We followed the recently developed recommendations for disproportionality analyses in detecting drug safety signals using case safety reports in pharmacovigilance (READUS-PV) to ensure transparent and clear reporting of scientific findings. The READUS-PV checklist is provided in Supplementary Table S2.

## 3 Results

### 3.1 Basic characteristics of upadacitinib-related adverse drug events in IBD treatment

From the third quarter of 2019 to the first quarter of 2024, this study gathered a total of 7,037,004 adverse event reports from the FAERs database. Among these, 37,822 reports identified upadacitinib as the primary suspect drug in adverse drug events (ADEs), with 1,917 of these reports specifically related to the treatment of inflammatory bowel disease (IBD) (Table 3). There was an increasing trend in the number of cases reported annually. Among all adverse events (AEs), females comprised a higher percentage (45.49%) compared to males (42.04%). Regarding age distribution, a significant portion (60.15%) of the data lacked age information, which limited a deeper understanding of the relationship between age and adverse events. Among the reports with specified age data, the most common age group was adults aged 18–65 years (30.88%), followed by the elderly aged 65 years and above (8.09%). Concerning severe adverse outcomes caused by upadacitinib, other serious outcomes (56.72%) were the most frequently reported, followed by hospitalizations (37.16%) and deaths (4.08%).

**TABLE 3:** Basic information on ADEs related to upadacitinib used in IBD from the FAERS database (2019 Q3-2024 Q1).

Factors	Case number	Case proportion
Year		
2019	2	0.1%
2020	6	0.31%
2021	11	0.57%
2022	399	20.81%
2023	1,135	59.21%
2024	364	18.99%
Gender		
female	872	45.49%
male	806	42.04%
unkown	239	12.47%
Age (years)		
<18	17	0.89%
18–65	592	30.88%
≥65	155	8.09%
Unknow	1,153	60.15%
Reported countries		
United States	1,599	83.41%
Japan	71	3.70%
Germany	51	2.66%
Other	196	10.22%
Reporter		
Consumer	1,127	58.79%
Physician	466	24.31%
Pharmacist	316	16.48%
Unknown	8	0.42%
Serious outcomes		
Hospitalization	437	37.16%
Death	48	4.08%
Life threatening	16	1.36%
disability	5	0.43%
Other serious	667	56.72%

### 3.2 Detection of signals related to upadacitinib

#### 3.2.1 Signal detection based on system organ class (SOC) level

**FIGURE 2:**
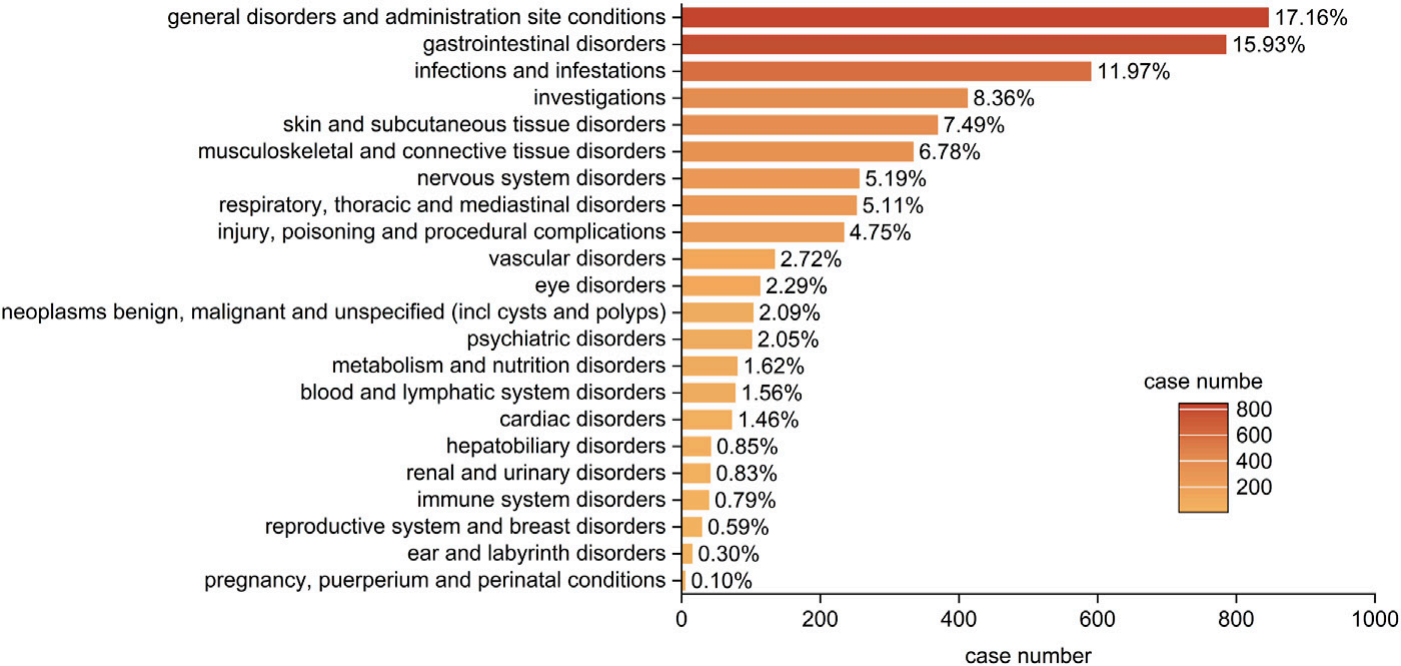
Bar plot illustrating the distribution of 22 system organ classes (SOCs) associated with adverse events of upadacitinib in IBD treatment. The percentage values represent the proportion of cases reporting adverse events in each SOC.

Statistical analysis revealed that adverse drug reactions (ADRs) caused by upadacitinib predominantly targeted 22 SOCs. The three most common systems affected were: general disorders and administration site conditions (n = 846, ROR 0.91, RPR 0.93M IC-0.11, EBGM 0.93), Gastrointestinal Disorders (n = 785, ROR 2.21, PRR 2.02, IC 1.01, EBGM 2.02), Infections and Infestations (n = 590, ROR 2.17, PRR 2.03, IC 1.02, EBGM 2.03) (Figure 2). These results partly correlate with the common adverse reactions listed in the drug’s package insert, indicating high credibility of the data. Notably, some of the SOCs involve significant adverse reactions not reflected in the drug’s package insert, including psychiatric disorders, renal and urinary disorders, and breast disorders. These findings suggest areas that may require further investigation and monitoring to better understand the full spectrum of upadacitinib’s safety profile.

#### 3.2.2 Signal detection based on preferred term (PT) level

At the PT level, this study utilized four algorithms to analyze adverse drug reactions and assess their compliance with various screening criteria, identifying 100 PTs. Based on the reporting odds ratio (ROR) algorithm, the top 50 PTs are shown in Table 4. The most commonly reported were semen discolouration, product residue present, and high density lipoprotein increased. In addition to side effects already mentioned in the package insert, this study also discovered rosacea, proctalgia, and polyp. Although these side effects occur less frequently, they presented with strong signal strength.

**TABLE 4:** The top 50 signal strength of adverse events of upadacitinib at the PTs level in FAERS database ranked by ROR.

System organ class (SOC)	Preferred term (PT)	Case reports	ROR (95% CI)	PRR (95% CI)	χ^2^	IC (IC025)	EBGM (EBGM05)
Reproductive system and breast disorders	Semen discolouration	5	363.83 (145.58, 909.26)	363.46 (144.67, 913.12)	1656.72	8.38 (7.17)	333.26 (154.86)
Investigations	Product residue present	52	70.12 (53.23, 92.37)	69.39 (52.74, 91.3)	3445.9	6.09 (5.7)	68.23 (54.18)
Investigations	High density lipoprotein increased	4	55 (20.5, 147.59)	54.96 (20.63, 146.44)	209.03	5.76 (4.49)	54.22 (23.74)
Skin and subcutaneous tissue disorders	Acne cystic	10	31.35 (16.81, 58.44)	31.28 (16.71, 58.57)	290.9	4.96 (4.1)	31.05 (18.44)
Investigations	Lipids increased	4	29.37 (10.98, 78.56)	29.34 (11.01, 78.18)	108.72	4.86 (3.59)	29.14 (12.79)
gastrointestinal disorders	gastric perforation	6	29.08 (13.02, 64.94)	29.04 (13, 64.86)	161.29	4.85 (3.78)	28.84 (14.72)
Gastrointestinal disorders	Malabsorption	9	26.84 (13.93, 51.73)	26.79 (14.03, 51.15)	222	4.73 (3.84)	26.62 (15.37)
Vascular disorders	Venous thrombosis limb	4	26.37 (9.86, 70.51)	26.35 (9.89, 70.21)	96.9	4.71 (3.44)	26.18 (11.5)
Infections and infestations	Genital herpes	3	26.26 (8.44, 81.76)	26.25 (8.42, 81.82)	72.38	4.7 (3.28)	26.08 (10.08)
Gastrointestinal disorders	Large intestinal haemorrhage	4	24.03 (8.99, 64.24)	24.01 (9.01, 63.97)	87.69	4.58 (3.31)	23.88 (10.49)
Infections and infestations	Appendicitis perforated	4	23.71 (8.87, 63.38)	23.69 (8.89, 63.12)	86.43	4.56 (3.29)	23.56 (10.35)
Infections and infestations	Large intestine infection	4	23.16 (8.66, 61.91)	23.14 (8.68, 61.66)	84.26	4.52 (3.25)	23.02 (10.11)
Gastrointestinal disorders	Proctitis	6	22.92 (10.27, 51.15)	22.89 (10.25, 51.13)	124.89	4.51 (3.44)	22.77 (11.63)
Investigations	Low density lipoprotein increased	10	20.82 (11.18, 38.78)	20.78 (11.1, 38.91)	187.33	4.37 (3.51)	20.68 (12.29)
Gastrointestinal disorders	Defaecation urgency	13	19.99 (11.58, 34.49)	19.94 (11.52, 34.52)	232.7	4.31 (3.55)	19.84 (12.57)
Eye disorders	Eye oedema	3	17.67 (5.68, 54.96)	17.66 (5.67, 55.04)	46.96	4.14 (2.72)	17.59 (6.81)
Skin and subcutaneous tissue disorders	Acne	128	16.81 (14.1, 20.05)	16.4 (13.75, 19.56)	1846.61	4.03 (3.78)	16.34 (14.1)
General disorders and administration site conditions	Adverse food reaction	3	16.49 (5.3, 51.25)	16.48 (5.29, 51.36)	43.43	4.04 (2.62)	16.41 (6.35)
Gastrointestinal disorders	Large intestine perforation	8	16.31 (8.14, 32.68)	16.29 (8.2, 32.35)	114.32	4.02 (3.07)	16.22 (9.07)
Gastrointestinal disorders	Mucous stools	9	16.21 (8.42, 31.22)	16.19 (8.48, 30.91)	127.73	4.01 (3.11)	16.13 (9.32)
Gastrointestinal disorders	Frequent bowel movements	41	16.05 (11.79, 21.84)	15.92 (11.63, 21.78)	571.48	3.99 (3.55)	15.86 (12.26)
Skin and subcutaneous tissue disorders	Rosacea	6	15.95 (7.15, 35.57)	15.93 (7.13, 35.58)	83.62	3.99 (2.92)	15.87 (8.11)
Hepatobiliary disorders	Portal vein thrombosis	3	15.02 (4.83, 46.69)	15.01 (4.82, 46.78)	39.09	3.9 (2.49)	14.96 (5.79)
Gastrointestinal disorders	Proctalgia	7	14.6 (6.95, 30.69)	14.58 (6.92, 30.71)	88.25	3.86 (2.86)	14.53 (7.81)
Infections and infestations	Gastric infection	5	14.4 (5.98, 34.66)	14.38 (5.95, 34.74)	62.04	3.84 (2.68)	14.33 (6.87)
Gastrointestinal disorders	Large intestine polyp	7	14.16 (6.74, 29.76)	14.14 (6.71, 29.78)	85.19	3.82 (2.81)	14.1 (7.57)
Gastrointestinal disorders	Gastrointestinal perforation	4	14.09 (5.28, 37.62)	14.08 (5.28, 37.52)	48.43	3.81 (2.54)	14.03 (6.17)
Infections and infestations	Cytomegalovirus colitis	3	14.07 (4.53, 43.73)	14.06 (4.51, 43.82)	36.27	3.81 (2.39)	14.02 (5.43)
Investigations	Blood cholesterol abnormal	5	13.71 (5.7, 33.01)	13.7 (5.67, 33.1)	58.68	3.77 (2.61)	13.66 (6.55)
Investigations	Inflammatory marker increased	7	12.87 (6.13, 27.05)	12.86 (6.11, 27.08)	76.3	3.68 (2.68)	12.82 (6.89)
General disorders and administration site conditions	Polyp	7	12.84 (6.11, 26.97)	12.82 (6.09, 27)	76.05	3.68 (2.67)	12.78 (6.87)
Infections and infestations	Herpes simplex	5	12.58 (5.23, 30.29)	12.57 (5.2, 30.37)	53.1	3.65 (2.49)	12.54 (6.01)
Infections and infestations	*Clostridium difficile* infection	25	12.33 (8.32, 18.28)	12.27 (8.29, 18.16)	258.14	3.61 (3.06)	12.24 (8.8)
Infections and infestations	Atypical pneumonia	3	12.26 (3.95, 38.09)	12.25 (3.93, 38.18)	30.91	3.61 (2.19)	12.22 (4.73)
Infections and infestations	Folliculitis	13	12.07 (7, 20.82)	12.04 (6.95, 20.84)	131.23	3.59 (2.83)	12.01 (7.61)
General disorders and administration site conditions	General symptom	3	11.94 (3.84, 37.1)	11.93 (3.83, 37.18)	29.97	3.57 (2.16)	11.9 (4.61)
Neoplasms benign, malignant and unspecified (incl cysts and polyps)	Rectal cancer	3	11.87 (3.82, 36.88)	11.86 (3.81, 36.96)	29.76	3.56 (2.15)	11.83 (4.58)
Gastrointestinal disorders	Diarrhoea haemorrhagic	9	11.83 (6.15, 22.77)	11.81 (6.19, 22.55)	88.8	3.56 (2.66)	11.78 (6.81)
Investigations	Mean cell volume increased	3	11.81 (3.8, 36.7)	11.81 (3.79, 36.81)	29.58	3.56 (2.14)	11.77 (4.56)
Investigations	Blood cholesterol increased	35	11.41 (8.18, 15.91)	11.33 (8.12, 15.81)	329	3.5 (3.02)	11.3 (8.55)
Vascular disorders	Embolism venous	3	10.9 (3.51, 33.86)	10.89 (3.49, 33.94)	26.89	3.44 (2.02)	10.87 (4.21)
Respiratory, thoracic and mediastinal disorders	Pulmonary thrombosis	11	10.46 (5.78, 18.91)	10.44 (5.8, 18.8)	93.66	3.38 (2.56)	10.41 (6.34)
Gastrointestinal disorders	Flatulence	39	9.68 (7.06, 13.27)	9.61 (7.02, 13.15)	300.47	3.26 (2.81)	9.59 (7.37)
Gastrointestinal disorders	Haematochezia	50	9.66 (7.31, 12.77)	9.57 (7.27, 12.59)	383.41	3.26 (2.86)	9.55 (7.56)
Respiratory, thoracic and mediastinal disorders	Respiratory symptom	3	9.45 (3.04, 29.35)	9.44 (3.03, 29.42)	22.6	3.24 (1.82)	9.42 (3.65)
Investigations	Blood triglycerides increased	8	9.34 (4.66, 18.7)	9.32 (4.69, 18.51)	59.33	3.22 (2.27)	9.31 (5.21)
Infections and infestations	Herpes virus infection	4	9.29 (3.48, 24.8)	9.29 (3.49, 24.75)	29.51	3.21 (1.95)	9.27 (4.08)
Infections and infestations	Abdominal abscess	3	9.18 (2.96, 28.52)	9.18 (2.95, 28.61)	21.81	3.2 (1.78)	9.16 (3.55)
Gastrointestinal disorders	Gastrointestinal pain	8	9.11 (4.55, 18.25)	9.1 (4.58, 18.07)	57.55	3.18 (2.24)	9.08 (5.08)
Eye disorders	Retinal detachment	6	9.02 (4.05, 20.11)	9.01 (4.03, 20.12)	42.64	3.17 (2.1)	8.99 (4.6)

Subgroup analyses revealed that in the elderly, the most reported AEs were pulmonary embolism, cataract, and sepsis (Table 5), while in the 18–65 age group, the most reported were acne, abdominal pain, and nasopharyngitis (Table 6).

**TABLE 5:** Signal strength of upadacitinib adverse events at PT level in FAERS, ranked by ROR for ages ≥65.

System organ class (SOC)	Preferred term (PT)	Case reports	ROR (95% CI)	PRR (95% CI)	χ^2^	IC (IC025)	EBGM (EBGM05)
Gastrointestinal disorders	Large intestine polyp	3	61.53 (19.69, 192.28)	61.01 (19.57, 190.15)	176.2	5.92 (4.5)	60.7 (23.4)
Respiratory, thoracic and mediastinal disorders	Pulmonary thrombosis	3	27.76 (8.9, 86.62)	27.53 (8.83, 85.8)	76.56	4.78 (3.36)	27.47 (10.6)
Investigations	Blood cholesterol increased	4	22.57 (8.42, 60.53)	22.32 (8.38, 59.47)	81.36	4.48 (3.2)	22.28 (9.76)
Respiratory, thoracic and mediastinal disorders	Respiratory disorder	3	18.61 (5.97, 58.03)	18.46 (5.92, 57.54)	49.48	4.2 (2.78)	18.43 (7.12)
Injury, poisoning and procedural complications	Upper limb fracture	3	17.56 (5.63, 54.76)	17.42 (5.59, 54.29)	46.38	4.12 (2.7)	17.39 (6.72)
Eye disorders	Cataract	6	11.53 (5.14, 25.86)	11.35 (5.18, 24.86)	56.67	3.5 (2.42)	11.34 (5.77)
Vascular disorders	deep vein thrombosis	3	9.78 (3.14, 30.49)	9.7 (3.11, 30.23)	23.43	3.28 (1.86)	9.7 (3.75)
Respiratory, thoracic and mediastinal disorders	Pulmonary embolism	5	9.17 (3.79, 22.18)	9.06 (3.82, 21.46)	35.86	3.18 (2.01)	9.05 (4.32)
Infections and infestations	Herpes zoster	3	6.98 (2.24, 21.74)	6.92 (2.22, 21.57)	15.21	2.79 (1.37)	6.92 (2.67)
Infections and infestations	Bronchitis	3	6.74 (2.16, 21)	6.69 (2.15, 20.85)	14.53	2.74 (1.32)	6.69 (2.58)
Infections and infestations	Sepsis	4	4.8 (1.79, 12.88)	4.76 (1.79, 12.68)	11.91	2.25 (0.98)	4.76 (2.09)

**TABLE 6:** Top 25 signal strengths of upadacitinib adverse events at PT level in FAERS, ranked by ROR for ages 18–65.

System organ class (SOC)	Preferred term (PT)	Case reports	ROR (95% CI)	PRR (95% CI)	χ^2^	IC (IC025)	EBGM (EBGM05)
Reproductive system and breast disorders	Semen discolouration	4	661.07 (230.46, 1896.28)	659.47 (228.84, 1900.43)	2279.25	9.16 (7.79)	571.67 (236.71)
Investigations	Product residue present	16	104.13 (63.27, 171.38)	103.14 (63.19, 168.36)	1580.45	6.65 (5.96)	100.74 (66.39)
Skin and subcutaneous tissue disorders	Acne cystic	5	39.44 (16.33, 95.27)	39.33 (16.28, 95.01)	185.07	5.28 (4.12)	38.98 (18.64)
Skin and subcutaneous tissue disorders	Rosacea	5	35.83 (14.84, 86.5)	35.72 (14.79, 86.29)	167.37	5.15 (3.98)	35.43 (16.95)
Gastrointestinal disorders	Proctitis	3	31.19 (10.01, 97.21)	31.14 (9.99, 97.06)	86.88	4.95 (3.53)	30.92 (11.94)
Gastrointestinal disorders	Malabsorption	3	26.84 (8.62, 83.59)	26.79 (8.6, 83.5)	74.03	4.74 (3.31)	26.63 (10.29)
Vascular disorders	Embolism	3	19.61 (6.3, 61.01)	19.57 (6.28, 61)	52.64	4.28 (2.86)	19.49 (7.54)
Gastrointestinal disorders	Large intestine polyp	3	18.48 (5.94, 57.5)	18.45 (5.92, 57.5)	49.31	4.2 (2.78)	18.38 (7.11)
Gastrointestinal disorders	Large intestine perforation	3	18.14 (5.83, 56.45)	18.11 (5.81, 56.44)	48.3	4.17 (2.75)	18.04 (6.98)
Skin and subcutaneous tissue disorders	Acne	40	15.43 (11.27, 21.13)	15.08 (11.02, 20.63)	524.86	3.91 (3.46)	15.03 (11.56)
Gastrointestinal disorders	Frequent bowel movements	17	15.38 (9.53, 24.82)	15.23 (9.51, 24.38)	225.42	3.92 (3.25)	15.18 (10.17)
Eye disorders	Retinal detachment	3	14.69 (4.72, 45.68)	14.66 (4.7, 45.69)	38.07	3.87 (2.45)	14.62 (5.66)
Gastrointestinal disorders	Proctalgia	3	14.49 (4.66, 45.06)	14.47 (4.64, 45.1)	37.48	3.85 (2.43)	14.42 (5.58)
Neoplasms benign, malignant and unspecified (incl cysts and polyps)	Colon cancer	4	12.77 (4.78, 34.11)	12.74 (4.78, 33.95)	43.15	3.67 (2.4)	12.7 (5.58)
Gastrointestinal disorders	Diarrhoea haemorrhagic	4	12.18 (4.56, 32.53)	12.15 (4.56, 32.37)	40.83	3.6 (2.33)	12.12 (5.33)
Metabolism and nutrition disorders	Malnutrition	3	12.04 (3.87, 37.42)	12.02 (3.86, 37.46)	30.22	3.58 (2.17)	11.99 (4.64)
Infections and infestations	Gastrointestinal infection	3	11.79 (3.79, 36.64)	11.77 (3.78, 36.68)	29.48	3.55 (2.13)	11.74 (4.54)
Infections and infestations	*Clostridium difficile* infection	8	11.55 (5.76, 23.16)	11.5 (5.79, 22.84)	76.52	3.52 (2.57)	11.47 (6.41)
Infections and infestations	Folliculitis	6	11.39 (5.1, 25.41)	11.35 (5.08, 25.35)	56.5	3.5 (2.43)	11.32 (5.78)
Investigations	Platelet count increased	4	11.37 (4.25, 30.36)	11.34 (4.26, 30.21)	37.62	3.5 (2.23)	11.31 (4.97)
Infections and infestations	Oral herpes	9	11.36 (5.9, 21.9)	11.31 (5.92, 21.6)	84.38	3.5 (2.6)	11.28 (6.52)
Gastrointestinal disorders	Defaecation urgency	3	11.1 (3.57, 34.52)	11.09 (3.56, 34.57)	27.46	3.47 (2.05)	11.06 (4.28)
Infections and infestations	Diverticulitis	7	10.07 (4.79, 21.17)	10.03 (4.76, 21.12)	56.79	3.32 (2.32)	10.01 (5.37)
Neoplasms benign, malignant and unspecified (incl cysts and polyps)	Basal cell carcinoma	4	9.64 (3.61, 25.75)	9.62 (3.61, 25.63)	30.84	3.26 (2)	9.6 (4.22)
Infections and infestations	Cytomegalovirus infection	5	8.79 (3.65, 21.16)	8.77 (3.63, 21.19)	34.34	3.13 (1.97)	8.75 (4.19)

### 3.3 Timing of adverse events

**FIGURE 3:**
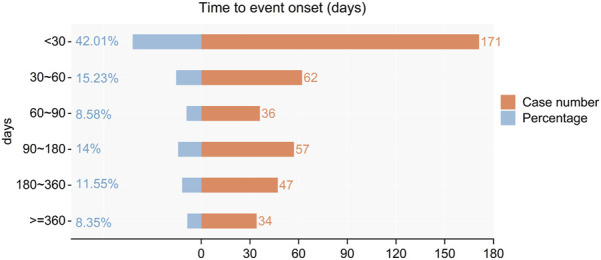
Time to event onset times.

The onset times of adverse events (AEs) related to Upadacitinib were extracted from the database. After excluding reports with inaccurate, missing, or unknown onset times, a total of 171 reports of Upadacitinib AEs with specified onset times were analyzed. The median onset time was 41.00 days (interquartile range [IQR] 10–141.5 days). As shown in Figure 3, the majority of AE cases (42.01%) occurred within 1 month of initiating Upadacitinib treatment. Notably, AEs can still occur 1 year after starting treatment, accounting for 8.35% of the cases.

## 4 Discussion

Upadacitinib is a small molecule inhibitor targeting the Janus kinase (JAK) pathway, which is involved in many immune-mediated inflammatory diseases. Consequently, the indications for upadacitinib include atopic dermatitis (Reich et al., 2021), rheumatoid arthritis (Genovese et al., 2018), psoriatic arthritis (Mease et al., 2021), non-radiographic axial spondyloarthritis (Deodhar et al., 2022), ankylosing spondylitis (van der Heijde et al., 2019), ulcerative colitis (Danese et al., 2022), and Crohn’s disease (Sandborn et al., 2020). As a novel oral small molecule treatment for inflammatory bowel disease (IBD), upadacitinib shows potential therapeutic advantages. However, it is crucial to monitor its real-world use and adverse events among IBD patients to ensure its safety and efficacy. This study systematically assesses the adverse reactions associated with upadacitinib by analyzing the FDA Adverse Event Reporting System (FAERS) database from the second quarter of 2019 to the first quarter of 2024, confirming some existing safety information and revealing new potential risks.

In the adverse events related to upadacitinib, the proportion of females was 45.49%, slightly higher than males at 42.04%. However, due to 12.47% of cases being unaccounted for, we cannot ascertain a credible gender ratio within these instances. It is noteworthy that the majority of adverse reaction reports (58.79%) were submitted by consumers rather than healthcare professionals. This could indicate that patients are more likely to report adverse reactions directly after using upadacitinib or reflect a deficiency in reporting by medical professionals.

According to disproportionality analysis, the most common and significant System Organ Classes (SOCs) such as “gastrointestinal disorders,” “infections and infestations,” and “vascular disorders” are consistent with safety data from labels and clinical trials. In the gastrointestinal disorders SOC, the most frequently reported adverse events were haematochezia, frequent bowel movements, flatulence, and defecation urgency. For infections and infestations, the most common were *Clostridium difficile* infections, followed by folliculitis, gastric infections, and herpes simplex.

With the aging of society, the number of elderly IBD patients is increasing, and our study found that the risk of thrombosis, including pulmonary thrombosis, pulmonary embolism, and deep vein thrombosis, is higher in the elderly, as is the risk of infections such as sepsis, oral candidiasis, and herpes zoster, related to the frailty and comorbidities of this population group. This suggests that upadacitinib should be used with caution in the elderly. The risk of thrombosis may be dependent on the duration of exposure, particularly in patients using it for 12 months or longer (Maqsood et al., 2022). Recent meta-analyses have shown that the risk of venous thromboembolism (VTE) may increase in patients with immune-mediated inflammatory diseases treated with JAK inhibitors compared to placebo or tumor necrosis factor inhibitors, although these findings did not reach statistical significance (Campanaro et al., 2023; Zhang et al., 2023). Given that the risk of VTE in IBD patients is significantly higher compared to the general population, further analysis of the potential link between JAK inhibitors and VTE risk is needed. All IBD patients should be screened for cardiovascular risk factors and risk stratification before using upadacitinib (Olivera et al., 2024).

In adults aged 18–65, acne is the most common side effect described for JAK inhibitors, including upadacitinib. It is usually reported as mild, self-resolving, or manageable with local treatments such as antibiotics, retinoids, or benzoyl peroxide (Mendes-Bastos et al., 2022). In clinical trials of upadacitinib, acne was shown to be dose-dependent, occurring almost exclusively in those taking 45 mg (Danese et al., 2022).

Our study indicates that the majority of cases occurred within the first 3 months after initiating Upadacitinib treatment (n = 269, 23.19%), particularly within the first month. This association may be due to the higher doses used during the initial 3-month induction period, as adverse reactions have been demonstrated to be dose-dependent (Nunez et al., 2023; Charles-Schoeman et al., 2024). However, many cases did not report specific times for adverse reactions (n = 753, 64.91%). Therefore, future clinical research on Upadacitinib should incorporate longer follow-up periods to more accurately observe its adverse drug reactions (ADRs).

Data on pregnant IBD patients are scarce, and current guidelines from the European Crohn’s and Colitis Organisation (ECCO) advise against using JAK inhibitors during pregnancy (Torres et al., 2023). Although no human studies have assessed the safety of upadacitinib in pregnancy, teratogenic effects have been observed in animal studies (Monfared et al., 2023). At the SOC level, there were also five cases related to pregnancy, puerperium, and perinatal conditions, indicating potential safety risks associated with the drug during pregnancy. Until new data are available, the use of this drug should be avoided during pregnancy.

Like all spontaneous reporting systems, FAERS has limitations such as underreporting, duplicate reports, missing data, and inaccuracies in reporting. These reports cannot establish a causal relationship between the drug and AEs or the drug’s safety. Nonetheless, the World Health Organization and the International Council for Medical Science consider spontaneous reporting system databases to be important sources of post-marketing safety surveillance, and their signal mining analysis can provide valuable insights for clinical safety medication. Furthermore, as upadacitinib has been used for a relatively short time in IBD, there is a potential for outcome bias, and more data should be gathered to further evaluate this.

## 5 Conclusion

This study, based on the FAERS system, has scientifically and systematically quantified the potential risks, onset times of adverse events (AEs), and the spectrum of safety signals associated with Upadacitinib treatment. It has identified semen discoloration, rosacea, and proctalgia as potential new indicators of adverse reactions to Upadacitinib. Furthermore, the analysis revealed variations in the prevalence of specific Preferred Terms (PTs) across different age groups; notably, a higher risk of thrombosis was observed in elderly patients, while younger individuals exhibited a greater propensity for reactions such as acne. These findings furnish valuable insights that can direct further research and enhance clinical practice concerning Upadacitinib, thereby improving its management and augmenting patient safety.

## Data Availability

The original contributions presented in the study are included in the article/Supplementary Material, further inquiries can be directed to the corresponding author.
