# Supplementary material for: Disproportionality analysis of upadacitinib-related adverse events in inflammatory bowel disease using the FDA adverse event reporting system
Source: Front Pharmacol. 2025 Feb 11;16:1436183. doi: 10.3389/fphar.2025.1436183 (PMC11851071; doi:10.3389/fphar.2025.1436183)
Supplement: Supplementary file 1 [file Table1.docx]

Supplementary Material

**Table S1**. The signal strength of ADEs of upadacitinib at the SOC level in FAERS database

| **System organ class** | **Case Reports** | **ROR (95% CI)** | **PRR (95% CI)** | **χ^2^** | **IC (IC025)** | **EBGM (EBGM05)** |
| --- | --- | --- | --- | --- | --- | --- |
| general disorders and administration site conditions | 846 | 0.91(0.85, 0.98) | 0.93(0.88, 0.99) | 6.04 | -0.11(-0.22) | 0.93(0.87) |
| gastrointestinal disorders | 785 | 2.21(2.05, 2.38) | 2.02(1.9, 2.14) | 436.65 | 1.01(0.9) | 2.02(1.89) |
| infections and infestations | 590 | 2.17(1.99, 2.37) | 2.03(1.88, 2.2) | 329.13 | 1.02(0.9) | 2.03(1.89) |
| investigations | 412 | 1.41(1.27, 1.56) | 1.37(1.24, 1.51) | 44.68 | 0.46(0.31) | 1.37(1.26) |
| skin and subcutaneous tissue disorders | 369 | 1.35(1.21, 1.5) | 1.32(1.2, 1.46) | 30.8 | 0.4(0.25) | 1.32(1.21) |
| musculoskeletal and connective tissue disorders | 334 | 1.28(1.15, 1.44) | 1.27(1.15, 1.4) | 19.65 | 0.34(0.18) | 1.27(1.15) |
| nervous system disorders | 256 | 0.67(0.59, 0.76) | 0.68(0.6, 0.76) | 40.49 | -0.55(-0.73) | 0.68(0.62) |
| respiratory, thoracic and mediastinal disorders | 252 | 1.09(0.96, 1.24) | 1.08(0.96, 1.21) | 1.75 | 0.12(-0.07) | 1.08(0.98) |
| injury, poisoning and procedural complications | 234 | 0.34(0.3, 0.39) | 0.37(0.33, 0.42) | 281.07 | -1.42(-1.61) | 0.37(0.33) |
| vascular disorders | 134 | 1.42(1.2, 1.69) | 1.41(1.18, 1.68) | 16.23 | 0.49(0.25) | 1.41(1.22) |
| eye disorders | 113 | 1.15(0.95, 1.39) | 1.15(0.96, 1.37) | 2.17 | 0.2(-0.07) | 1.15(0.98) |
| neoplasms benign, malignant and unspecified (incl cysts and polyps) | 103 | 0.48(0.4, 0.59) | 0.49(0.4, 0.6) | 55.96 | -1.02(-1.3) | 0.49(0.42) |
| psychiatric disorders | 101 | 0.35(0.28, 0.42) | 0.36(0.3, 0.44) | 122.94 | -1.48(-1.76) | 0.36(0.3) |
| metabolism and nutrition disorders | 80 | 0.81(0.65, 1.01) | 0.81(0.65, 1) | 3.44 | -0.3(-0.61) | 0.81(0.68) |
| blood and lymphatic system disorders | 77 | 0.88(0.7, 1.1) | 0.88(0.71, 1.09) | 1.32 | -0.19(-0.51) | 0.88(0.73) |
| cardiac disorders | 72 | 0.71(0.56, 0.9) | 0.72(0.57, 0.91) | 8.28 | -0.48(-0.82) | 0.72(0.59) |
| hepatobiliary disorders | 42 | 1(0.74, 1.35) | 1(0.75, 1.34) | 0 | 0(-0.43) | 1(0.77) |
| renal and urinary disorders | 41 | 0.42(0.31, 0.57) | 0.42(0.31, 0.57) | 32.94 | -1.24(-1.68) | 0.42(0.33) |
| immune system disorders | 39 | 0.66(0.48, 0.91) | 0.66(0.48, 0.9) | 6.74 | -0.59(-1.04) | 0.66(0.51) |
| reproductive system and breast disorders | 29 | 0.94(0.65, 1.36) | 0.94(0.65, 1.36) | 0.11 | -0.09(-0.61) | 0.94(0.69) |
| ear and labyrinth disorders | 15 | 0.71(0.43, 1.18) | 0.71(0.43, 1.18) | 1.77 | -0.49(-1.2) | 0.71(0.47) |
| pregnancy, puerperium and perinatal conditions | 5 | 0.28(0.12, 0.68) | 0.28(0.12, 0.68) | 9.09 | -1.82(-2.97) | 0.28(0.14) |

**Table S2**. The READUS-PV checklist

| **Section and topic** | **Item #** | **Checklist item** | **Location where item is reported** |
| --- | --- | --- | --- |
| **Title** |  |  |  |
|  | *1a* | *If disproportionality analyses are a prominent component of the published study, the study should be identified as a “disproportionality analysis”. The type of data and name of the database(s) should be specified.* | *Title* |
|  | *1b* | *Report the name of adverse event(s) and/or drug(s) under study, when applicable.* | *Title* |
| **Introduction** |  |  |  |
| Background | *2a* | *Describe the drug(s) and its utilization, the nature of the adverse event(s) under study and its frequency, and the existing knowledge on the drug-event combination.* | *Line66-72* |
|  | *2b* | *Specify the rationale for performing the analysis, e.g., as part of routine pharmacovigilance, to investigate an overall safety profile, or to assess a pre-specified hypothesis.* | *Line73-74* |
|  | *2c* | *Explain why ICSR databases and disproportionality analysis are suitable to fill the knowledge gap.* | *Line74-76* |
| Objectives | *3* | *State specific objectives, identifying the adverse event(s), the drug(s), and the reference group, including any pre-specified hypothesis, if applicable.* | *Line77-81* |
| **Methods** |  |  |  |
| Study design | *4a* | *Identify the study (i.e., “disproportionality analysis”) and the type of data used (e.g., “individual case safety reports”).* | *Line84-87* |
|  | *4b* | *Provide an outline of the entire study design, including primary and sensitivity analyses performed, and other designs such as case-by-case analysis or literature review.* | *Figure 1* |
| Data description, access, and pre-processing | *5a* | *Specify the name of the database(s), the database(s) custodian, and the coverage. Specify the type/number of drugs included within the database and the thesaurus, taxonomies, or ontologies used for coding drugs and events.* | *Line85-90* |
|  | *5b* | *Specify the extraction dates and describe and justify all choices used for data pre-processing, including any data transformation or exclusion, if appropriate.* | *Line87-95* |
| Variables definition | *6a* | *Describe the study population, including any restriction.* | *Line99-102* |
|  | *6b* | *Describe the nature and the meaning of key variables assessed in the work.* | *Line124-127* |
|  | *6c* | *Specify and justify any grouping of drugs or events. For drugs, specify and justify whether active ingredients/trade names/salts were considered and/or the selected role.* | *Line96-97* |
|  | *6d* | *Describe any additional data source used, the type of data, and how they interact with ICSRs.* | *none* |
| Statistical methods | *7a* | *Present any descriptive analysis performed, specifying variables investigated, statistical tests, and significance thresholds.* | *Line99-103* |
|  | *7b* | *Describe the measure(s) selected for the disproportionality analysis including any threshold used to identify signals of disproportionate reporting. Explain the reason for this choice if applicable.* | *Line107-122* |
|  | *7c* | *Clearly describe any sensitivity analysis and any tool to control confounding, including any restriction, subgroup, stratification, adjustment, or interaction.* | *none* |
|  | *7d* | *Specify the variables and methods used for the case-by-case analysis, including any algorithm or criteria used to assess causality, if performed.* | *none* |
|  | *7e* | *Specify any statistical methods used for other data sources.* | *none* |
| **Results** |  |  |  |
| Participants | *8a* | *Specify the number of individual case safety reports included at each stage, including reasons for exclusion.* | *Line134-145* |
|  | *8b* | *Provide key demographic and clinical characteristics of cases, if possible comparing cases with any appropriate reference group.* | *Table 3* |
| Disproportionality analysis | *9* | *Present all results including confidence intervals. Present also results of sensitivity analyses, if performed.* | *Table 4& table5& table6* |
| Case-by-case analysis | *10* | *Present the case-by-case analysis of key variables. Present the causality assessment, if applicable.* | *Line 169-175* |
| **Discussion** |  |  |  |
| Key results | *11* | *Discuss key results with reference to study objectives and contextualize them within the current literature and other consulted sources. Clearly discriminate between expected reactions and emerging safety signals.* | *Line187-244* |
| External validity | *12a* | *Discuss the external validity of the results to the general population.* | *Line212-244* |
|  | *12b* | *Discuss the potential relevance of results in clinical practice* | *Line212-244* |
|  | *12c* | *Propose further study designs if applicable* | *none* |
| Limitations | *13* | *Present general limitations, making clear that disproportionality analysis alone cannot prove causation or measure incidence, and specific limitations, including confounding and reporting bias and efforts to mitigate them.* | *Line245-252* |
| **Declarations** |  |  |  |
|  | *14a* | *Provide the source of funding/sponsorship and the role of the funders/sponsors for the present study and for any original study on which the present article is based.* | *Line275-276* |
|  | *14b* | *Clearly identify potential commercial and intellectual conflicts of interest (e.g., link to any drug/event investigated, whether financial, legal action, or software used).* | *Line278* |
|  | *14c* | *Declare any institutional approval needed or granted in the investigation.* | *none* |
|  | *14d* | *Include a statement on data availability, code availability (including the version of the statistical software used), and protocol registration.* | *Line264-266* |
